# Supplementary material for: The Deployment of a Newly Developed Proximal Release-Type Colonic Stent Is Feasible for Malignant Colorectal Obstruction near the Anal Verge: A Single-Center Preliminary Study
Source: J Clin Med. 2022 Mar 17;11(6):1675. doi: 10.3390/jcm11061675 (PMC8955481; doi:10.3390/jcm11061675)
Supplement: Supplementary file 1 [file jcm-11-01675-s001.zip › jcm-1607990-supplementary.pdf]

Supplementary Material Table S1. Details of four BTS cases

Age/sex	65, M	77, F	70, F	77, F
Etiology of colorectal obstruction	Primary colorectal cancer	Primary colorectal cancer	Primary colorectal cancer	Primary colorectal cancer
Duration from diagnosis to SEMS placement, days	2	0	0	3
ECOG PS score	0	0	0	1
CROSS score	1	0	1	3
Location	S	S	S	S
Distance from the anal verge, cm	8	7	9	6
Stricture length, cm	4	10	3	10
Procedure time, min	16	35	75	29
Duration from SEMS placement to the start of water intake, days	3	5	1	1

Duration from SEMS placement to the start of oral intake, days	4	13	3	2
Adverse event	-	-	Migration	-
Duration from SEMS to surgery, days	11	144	26	28
Surgical procedure	Colectomy with primary anastomosis	Colectomy with primary anastomosis	Colectomy with primary anastomosis	Colectomy with primary anastomosis
Stoma creation	-	-	-	Diverting*
Postoperative complication	-	-	-	Anastomotic leakage*
Duration of hospitalization, days	8	25	12	21
Overall survival, days	344	276	222	49
	Death	Alive	Alive	Alive
Cause of death	Pneumonia	-	-	-

* Emergency reoperation with diverting stoma

Supplementary Material Table S2. Details of four PAL cases

Age/sex	64, M	67, F	79, M	80, F
Etiology of colorectal obstruction	Gastric cancer	Anastomotic recurrence	Pancreatic cancer	Gastric cancer
Duration from diagnosis to SEMS placement, days	539	935	1115	18
PS score	2	0	1	1
CROSS score	2	3	1	1
Location	Rb	Anastomosis of the sigmoid colon	Rb	Ra
Distance from the anal verge, cm	2	4	4	5
Stricture length, cm	4	4.3	4	4
Procedure time, min	14	17	11	7
Duration from SEMS placement to the start of water intake, days	1	1	1	1
Duration from SEMS placement to	3	2	4	40 (due to advanced-stage

the start of oral intake, days				gastric cancer)
Adverse event	-	-	-	-
Overall survival, days	128	420	99	62
	Dead	Dead	Alive	Alive
Cause of death	Cancer progression	Cancer progression	-	-
